# COVID 19: Frontline experience at a tertiary care hospital in UK

**DOI:** 10.7189/jogh.10.010356

**Published:** 2020-06

**Authors:** Faisal Bashir Chaudhry, Samavia Raza

**Affiliations:** 1 Acute Medicine Department, Royal Stoke University Hospital, University Hospital North Midlands NHS Trust, Stoke on Trent, Staffordshire, UK; 2 Imaging Directorate, Royal Stoke University Hospital, University Hospital North Midlands NHS Trust, Stoke on Trent, Staffordshire, UK

Health systems around the world are already working at full stretch and inherently under prepared to face a global calamity. The COVID 19 pandemic has all the ingredients of a stressful situation for health care providers. We share our experience of working at the front door of a busy tertiary care hospital in United Kingdom, at the peak of pandemic. We have suggested some helpful guides in service development, pointing out how we tackled increased workload of a contagious disease and how we established standard operating procedures in an ever-evolving situation. To minimise the spread of the disease, we developed a triage system and a separate pathway at the front door for suspected COVID 19 patients with a dedicated Resus and Majors area capable of providing level 2 care to 30 patients at one time. We have also highlighted the factors that had a negative impact on our efforts and morale while we battled with coronavirus, including sluggishness in ramping up testing capabilities and scandals surrounding procurement and supply of personal protective equipment to health care workers. We have also briefly discussed resultant psychological impact of the pandemic on our staff and suggested strategies that are being used locally. We hope that our account will help in future, if any similar crisis were to emerge and help those who are following us in the curve in the current pandemic. We also hope that discussing the problems faced by health services facing the pandemic would enable policy makers to direct resources towards key areas in a timelier manner, which will lead to a better prepared health system for everyone in future.

There is no denying that COVID-19 pandemic has all the ingredients for a highly stressful experience especially at the front door of an acute hospital. We work in a 1250 bedded tertiary care hospital in North of midlands in United Kingdom. The trust employees over 10 000 staff and provides acute hospital services to approximately 900 000 people living in Staffordshire, South Cheshire and Shropshire. We had our first confirmed case of coronavirus in the first week of March. Since then around 1500 patients have tested positive for SARS-COV-2 virus in Staffordshire through RT-PCR analysis of nasal and oro-pharyngeal swabs, with around 500 presenting to our hospital alone. In keeping with the national guidelines, we are only testing patients that are too unwell to be managed at home and need hospital admission [1]. Those who are well enough to be discharged from front door are not tested and are advised to self-isolate and monitor their symptoms at home. Among those who have been admitted to hospital in last six weeks, more than half of them have been successfully discharged home. As of 25th of April, 167 cases of confirmed COVID 19 have died in our hospital [2]. The figures are dynamic and subject to change by the time of publication.

Health systems across the globe, stretched to their limit in best of times, are now facing unprecedented challenges. This in turn has taken its toll on the physical and mental well-being of health care workers on the front line [3]. We share our experiences in the hope that this will help guide policy making and devising standard operating procedures in the future particularly for countries that are behind the curve in spread of pandemic.

A commendable initiative by most hospitals in our region was early clear demarcation of separate pathway for suspected COVID-19 cases. We labelled this pathway RED (respiratory emergency department). All other patients follow the Green pathway. Triage process to identify the appropriate pathway for a patient starts at the ambulance reception/triage area. Any patient with symptoms suggestive of COVID-19, including but not limited to fever, cough and shortness of breath of new onset, is directed to RED pathway. We have converted Paediatric Resus into RED Adult Resus, with 10 monitored beds and ability to provide level 2 and interim level 3 care. Old Adult Resus has been maintained as Green, as it has the capacity to accommodate trauma patients. Less unwell patients and step downs are directed to ED Majors area which is now dedicated to COVID-19 patients, comprising of 30 beds with ability to provide level 2 care in addition to RED Resus. Green Majors area has been transferred to Clinical decisions unit, an area previously being used for same day or overnight stable patients. Responsibility for same day care and stable overnight patients has been transferred to Acute Medical unit, which has been kept free from COVID-19 cases.

To minimise the number of staff being exposed to COVID-19 we have altered our approach to patient interaction and clerking. Traditionally a junior doctor would examine a patient and then discuss with a senior colleague who would then re-examine as second contact. We have established first contact now to be with a senior clinician. This has also saved precious Personal Protective Equipment (PPE) resources.

In this stressful situation, while some guidelines have been helpful, many have caused confusion and anxiety. There has been concern among the health care staff over the difference between national guidelines for PPE and those recommended by the World Health Organisation [4]. A highly awaited update to the initial substandard PPE guidelines was finally published on 2nd April 2020 by NHS leaders and UK government; probably a month too late for the staff that had either died or had contracted corona virus by that time only to succumb to it later. By the 5th week of this pandemic reaching the UK, although government only confirmed 49 verified deaths of NHS staff, sources claim the number of health care fatalities to be more than a hundred [5]. Over the years, due to budget constraints emergency stockpiles of PPE have dwindled and gone out of date, with many of us having to use PPE labelled past expiration dates [6]. Even the new guidelines are clearly devised keeping in mind the existing stocks rather than protection of staff as priority. Trying to sell these recommendations as data driven is only going to undermine trust between expert bodies and staff. We are working in areas with suspected as well as confirmed cohorts of COVID patients, wearing thin plastic Aprons and short gloves, leaving the whole of our arms exposed, despites studies clearly indicating that corona virus survives on skin and cloth. Inadequacy in this regard has had an adverse impact on staff morale. Amid this, implying that health care workers are responsible for wasting precious PPE, did not help [7]. Adding fuel to fire are the news of essential PPE being exported to Europe despite desperate shortage at home [8]. The potential of COVID-19 turning into a global catastrophe was recognised as early as January 2020, when one of the most reputable journals in health care, published a study comparing the novel corona virus outbreak with Spanish flu pandemic of 1918 [9]. The study advised that there was no room for complacency and time to act was now. In the following five weeks, response from governments was sluggish and precious time was wasted that could have been used in boosting the supplies of PPE and ramping up testing capacity. It is now becoming clear that contrary to the official stance, the country was in a poor state of readiness to face a pandemic [10]. A thorough official inquiry in coming days is inevitable and much will be learnt from its outcome.

**Figure:**
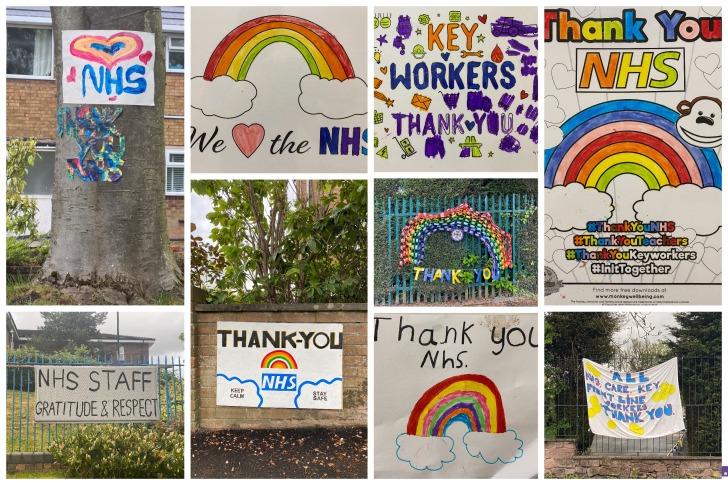
Photo: from the collection of Faisal Bashir Chaudhry (used with permission).

The recommendations from World Health Organisation were to test at a massive scale [11]. The lack of capacity in the initial weeks of pandemic meant that only a limited number of patients could be tested and the centres offering RT-PCR tests were few and far between [12]. We had to send samples off to remote site, resulting in a lag of 72 hours in getting the result. This left both the clinician and the patient in a limbo. By beginning of April, in-house testing facility was established in our trust and neighbouring hospitals, which has been a massive help for inpatient management. Still the overall number of patients being tested remains quite low with a massive gap between promised number of tests and actual capacity of testing. On 25 March 2020, the UK Prime Minister revealed aim to reach 250 000 tests per day. But we are far from reaching a more realistic 100 000 tests target by the end of April set by Health Secretary on 2 April 2020. Another important reason for the low number of tests is the obsolete guidelines which still recommend only sick symptomatic patients being admitted to hospital to be tested, with no mention of cases with mild symptoms or contact tracing. Consequently, despite the testing capacity reaching 40 000, actual number of tests peaked at 22814 on 21 April 2020 [13]. Staff testing is essential not only to enable essential work force to safely return to work but also to make the staff feel reassured and supported. It only arrived towards second half of April. The website that was launched for key workers to book tests for themselves, like all the promises, broke down within hours of its launch due to significant demand [14].

No amount of resilience training prepares one for the real-life situation faced in a crisis like this. Telling a daughter over the phone about her elderly father who is going to pass away in hospital, with her not being able to visit him, takes a toll. Supporting a colleague on a busy shift, whose family member has developed symptoms abroad, while having nightmares about your own, is a test of nerves. To cope with increased workload on the front door, we developed a 12-hour shift pattern for all medical personnel. This has ensured adequate staffing numbers with cushion for staff sickness. It must be appreciated that this is a marathon, not a sprint. Burn out as a result of constant physical and psychological stress is a real concern [15]. Feeling stressed in this unprecedented time is normal and by no means a reflection of inadequacy in one’s skills or ability. Different trusts are offering varying strategies like rest breaks, lunch boxes and letters of gratitude and support for employees. Staff support and counselling service hotline are also available. NHS England and NHS improvement (NHSE/I) have compiled a few useful mental health and well-being resources, which are all being offered free of charge to NHS staff. British Psychological Society has published guidelines and hosting webinars to discuss the risks that managing the pandemic brings for the psychological well-being of staff [16].

There is a real fear of contracting the virus among health care workers and many are concerned for the safety and well-being of their family, who may belong to a more vulnerable cohort. Some workers have chosen to isolate themselves from family to minimise risk of transmission but that has its own adverse psychological impact. We have been changing in our offices, garages, porches and going straight to shower, if not already done so at hospital. Personal devices like mobile phones, glasses and badges can act as vectors of transmission and should be cleaned at the end of every shift with appropriate sterilising solution.

Public response towards NHS staff has been heart-warming. On leaving the hospital, we see numerous messages of support displayed on walls and windows. Every Thursday people open their windows to clap and cheer to express their gratitude for the health workers. People are keen to help and support the NHS and nearly 1 million have signed up as volunteers [17]. There is also a huge spike in the donations to NHS charities. However, the opinion about donations is divided and many argue that the health service should not be dependent on handouts. The pandemic has put the National Health Service in the spotlight, not only its strengths but also its short-comings. One can expect that these issues which have been uncovered will be addressed going forward and the NHS will emerge stronger in many ways after COVID-19.
